# Information Technology Support for Clinical Genetic Testing within an Academic Medical Center

**DOI:** 10.3390/jpm6010004

**Published:** 2016-01-20

**Authors:** Samuel Aronson, Lisa Mahanta, Lei Lei Ros, Eugene Clark, Lawrence Babb, Michael Oates, Heidi Rehm, Matthew Lebo

**Affiliations:** 1 Partners HealthCare Personalized Medicine, 65 Landsdowne Street, Cambridge, MA 02139, USA; lmahanta@partners.org (L.M.); lhros@partners.org (L.L.R.); ehclark@partners.org (E.C.); lbabb@geneinsight.com (L.B.); mfoates@partners.org (M.O.); hrehm@partners.org (H.R.); mlebo@partners.org (M.L.); 2 Partners HealthCare Research Computing, 65 Landsdowne Street, Cambridge, MA 02139, USA; 3 Department of Pathology, Brigham & Women’s Hospital, Boston, MA 02115, USA; 4 Department of Pathology, Harvard Medical School, Boston, MA 02115, USA; 5 The Broad Institute of Massachusetts Institute of Technology and Harvard, Cambridge, MA 02142, USA

**Keywords:** genetic testing process, information technology support, LIMS, LIS, GeneInsight, GIGPAD

## Abstract

Academic medical centers require many interconnected systems to fully support genetic testing processes. We provide an overview of the end-to-end support that has been established surrounding a genetic testing laboratory within our environment, including both laboratory and clinician facing infrastructure. We explain key functions that we have found useful in the supporting systems. We also consider ways that this infrastructure could be enhanced to enable deeper assessment of genetic test results in both the laboratory and clinic.

## 1. Introduction

The clinical genetic testing process within an academic medical center involves multiple different groups. Clinicians identify the need for tests and order them. Then, laboratory technicians receive these orders with associated samples and perform the requested assays. The results of these assays are sent to laboratory personnel responsible for interpreting the results and ultimately producing a report that is signed out by a board certified professional. This report is then returned to the clinician who is responsible for assessing how the results should affect the patient’s care. The clinician shares the assessment with the patient and also stores and manages the results over time [1].

**Figure 1:**
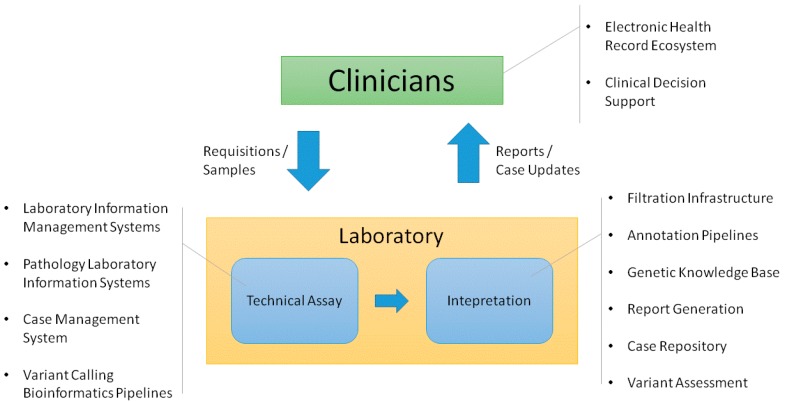
System functionality required to support the clinical genetic testing process flow.

Each of the groups involved in this process requires information technology (IT) support to perform its function in a high quality, efficient manner. This often involves establishing deep support for specific processes. Multiple systems may be needed to meet a group’s needs. In this context, it is important to determine how systems should be integrated across the workflow. The quality of the overall clinical process depends on data transferring in a robust, consistent manner. Figure 1 shows the infrastructure that we have found necessary to support these process flows in the germline genetic testing environment. There are multiple ways this functionality can be implemented. While a complete review of different methods is beyond the scope of this article, we will describe the infrastructure that we have established to support the clinical germline genetic testing processes implemented by our Laboratory for Molecular Medicine (LMM).

## 2. Clinician Facing Infrastructure

The electronic health record (EHR) is the basis for most clinical IT support. However, the EHR often has to be extended to provide in-depth support for a particular area or function [2]. As a result, it is useful to think in terms of an EHR ecosystem that includes other systems linked to the EHR. We have found that the clinical genetic IT support needed most by clinicians includes helping them receive and manage genetic test results as well as enabling them to stay up to date on changing genetic knowledge.

**Figure 2:**
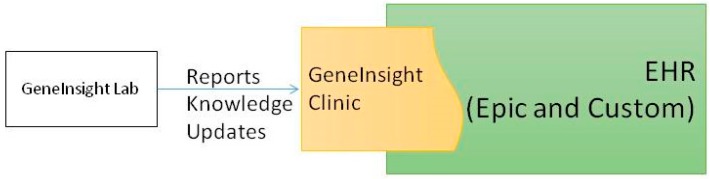
Clinician facing infrastructure.

Our institution is in the process of transitioning from a custom built EHR to Epic (Epic Systems, Verona, WI, USA). We have integrated an external application we created, GeneInsight Clinic [3], with both of these EHRs to provide genetics support for our clinicians (Figure 2). GeneInsight Clinic provides clinicians access to easily view genetic test results that have been executed on a patient by our LMM and the potentially meaningful variants that were identified by these tests by incorporating structured data delivered by the molecular genetics laboratory. Users can then drill down into the interpretation of those variants that were assessed relative to the indication for testing. It also provides clinicians with alerts when one of these interpretations is updated by the reporting lab in a potentially clinically significant way [4]. We also recognize the need for additional forms of clinical decision support (CDS) [5] including rules being developed by the National Academy of Medicine’s Display and Integrating Genetic Information Through the EHR Action Collaborative (DIGITizE AC) organized under the genomics roundtable. We have not yet leveraged Substitutable Medical Applications and Reusable Technology (SMART) [6] apps to extend our clinical genetic displays, but we believe this platform has great potential and are investigating doing so.

Currently, order and phenotypic information is transferred to the laboratory via paper requisition forms. An electronic order entry system could provide far deeper support, especially for exome and genome based assays. However, there is a challenge in that our laboratory receives orders from around the world. An electronic order entry system would need to be broadly accessible to have the maximum impact and ideally integrated with as many EHRs and laboratory information systems (LIS) as possible. In advance of such functionality becoming available, we have begun to investigate alternate approaches focused on providing the laboratory with access to EHR data when tests are ordered for patients within our environment.

## 3. Infrastructure Supporting the Technical Assay

### 3.1. The Technical Assay Workflow

**Figure 3:**
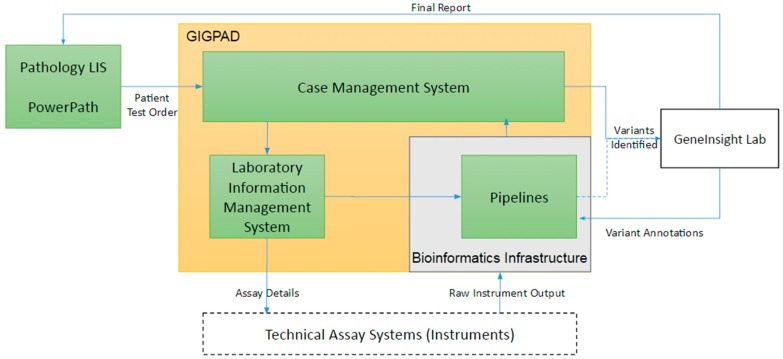
Infrastructure supporting the technical assay.

The technical workflow for each assay functions to identify variants present in the patient’s DNA associated to that test. From an IT perspective, it is important to support both the development of these tests and their execution for each patient. Developing tests often involves designing primary assays that broadly assess DNA either within regions of interest or across the whole exome or genome. Often, many technical platforms are required for a clinical test to be complete due to a few factors: (1) technical limitations of the assay requires an alternate platform, (2) confirmation of variants are required using an orthogonal technology, and (3) additional assays are required for completeness (*i.e.*, “fill-in” of the specific regions defined within the test). For all of these types of assays, bioinformatic pipelines are needed to process raw instrument output and convert it to variant calls and completeness and coverage of the tested region. In addition to data management, support is also needed to manage the large inventory of oligonucleotides (oligos) required to perform the DNA enrichment and isolation that underlie these assays. Once assays transition into production, laboratories require workflow support to track patients’ test details, manage experiment batch details, organize bioinformatics pipeline runs, record laboratory quality controls, store technical reviews, and consolidate information into case level summaries. Our support consists of three primary systems that are interconnected: PowerPath Pathology LIS, our Gateway for Integrated Genomics Proteomics Applications and Data (GIGPAD) system and our bioinformatic pipeline infrastructure (Figure 3). GIGPAD in turn serves as enterprise infrastructure that incorporates our Laboratory Information Management Systems (LIMS). We designed GIGPAD so that each lab can make an independent decision whether to custom build LIMS or buy a commercial solution. Either choice can be integrated into the GIGPAD “superstructure” to support coordinated process flows and piggyback on GIGPAD’s interfaces to other systems. (We use the term LIS to refer to enterprise pathology systems and the term LIMS to refer to systems focused on managing specific wet bench workflows surrounding instruments.) GIGPAD also contains a Case Management System (CMS).

### 3.2. The Use of Pathology Laboratory Information Systems

Pathology LISs can provide many important functions for clinical genetic laboratories. We use our hospital’s LIS, Sunquest PowerPath, to manage the initial stage of our test execution process beginning with sample accessioning. In sample accessioning, both specimen details and case data are entered. PowerPath is integrated with our organization's enterprise master patient index (EMPI) that allows us to assign identification numbers to patients that ease integrations across systems including to our EHR. In addition to accessioning and critical systems integrations, PowerPath provides support for report finalizations and billing processes. Multiple other Pathology LIS solutions are marketed by companies including Epic, Cerner (Cerner Corporation, Kansas City, MO, USA), and Soft Computer (SCC Soft Computer, Clear Water, FL, USA).

### 3.3. Case Management System (CMS)

Clinical laboratories providing complex molecular testing require support to manage patient testing that involves iterative assays with multiple different platform and analytical instrument integrations. The CMS functionality within our GIGPAD system manages the relationships between assays associated with each case, the experiment batches that are run for each case, and the management and technical interpretation of data generated from those assays. CMS tracks which assays are required in order to complete each case and orchestrates the performance of these assays. To do so, it relies on test definitions that define what regions of DNA should be assayed in each test. CMS communicates with individual LIMS modules to queue samples for processing. It also communicates with bioinformatic pipelines to receive and react to results by calling for fill-in or confirmatory assays as needed. In addition to managing the assays and corresponding reactions that are required for a patient, CMS also tracks the quality control metrics of each experiment batch. If any quality control metrics are tracked as “failed”, CMS will prevent the data associated to the experiments from being used for any downstream processes. Ultimately, when a case is progressed to a “Lab Complete” status in CMS, all the high quality results are released to GeneInsight Lab (see below) for interpretation and reporting.

### 3.4. Laboratory Information Management Systems (LIMS)

A combination of LIMS modules, spreadsheet templates, and excel macros manage the wet bench workflow through data generation on the instruments for clinical testing. At present, our clinical genetic testing process relies on custom built Sanger sequencing and Next Generation Sequencing (NGS) LIMS modules. The NGS LIMS in combination with spreadsheet templates supports the library preparation process and mapping of samples to experiment runs. The Sanger LIMS manages the process surrounding instrument run configuration including plate definition and loading on the machine. Once the data is generated and the instrument run is complete, GIGPAD also has functionality to extract data from instrument computers and deliver it to bioinformatic pipelines.

For many wet bench processes, the laboratory evolves more quickly than IT can provide robust solutions, whether custom built or purchased. The rapid pace of evolving technologies employed in molecular testing requires a balance of IT solutions that are robust and reliable, as well as flexible, allowing for modifications and rapid updates. This balance is a constant challenge that is continually revisited when determining the appropriate solution for laboratory needs.

### 3.5. Oligo Management

In many ways, oligos provide the foundation for current genetic testing processes. Oligos are synthetically manufactured nucleotides that are designed to target and enrich specific regions within the genome for downstream analysis. The mechanism through which they are designed and applied is determined by the technology and application being employed in the clinical test. Some examples of oligos include primers, baits, and probes. Each type of oligo can have a number of different and unique aspects including length, additional dyes, and variable bench conditions. Each of these variables must be captured for accurate experiment setup within the clinical workflow. For example, Sanger sequencing requires primers to target specific regions of DNA for PCR amplification to detect potential disease causing variants. In order for the experiment to work, any specific experimental conditions that were assigned during validation need to be applied. With the increasing complexity of genetic testing, databases are needed to track the designs and modifications that have been produced and their status in the validation process in order for testing to proceed robustly. We built an Oligo Management System (OMS) to support these functions that is integrated with CMS to assist the laboratory in choosing the best oligo for each assay and removing oligos from the production process that fail validation.

### 3.6. Variant Calling Bioinformatic Pipelines

The identification of patient genomic information is at the heart of molecular genetic testing, whether via Sanger sequencing, NGS, or copy number assessment. In each of these contexts, bioinformatics algorithms are used to transform the raw data generated by the instruments into data values that can be evaluated and interpreted. Our laboratory uses a standard reference genome (currently GRCh37) for consistent mapping across all of our technologies and assays. Specific differences from the reference genome are determined and highlighted, as well as information related to the quality and completeness of the assay. The data is further annotated using GeneInsight to be put into the correct clinical context; this includes determining the gene and transcripts, HGVS nomenclature, and, if available, previously determined pathogenicity associated with the variant. To ensure continuity, robustness, and repeatability, these algorithms are wrapped into IT pipeline frameworks that are then validated by the laboratory. Ideally, these pipelines not only process the data, but also manage computational resources, appropriately provide quality checkpoints, and track the data through the process to completion. We built a custom solution, Advanced Sequencing Automated Pipeline (ASAP), when first launching clinical NGS sequencing in 2011. This pipeline allows our bioinformaticians to manage the assays in a robust manner with minimal oversight. ASAP uses GATK best practices for alignment and variant calling of SNVs and indels [7,8], and custom scripts for annotation, copy number variation (CNV) calling [9], determining coverage, and triggering regions requiring Sanger follow-up.

## 4. Infrastructure Supporting Interpretation

### 4.1. The Interpretation Workflow

**Figure 4:**
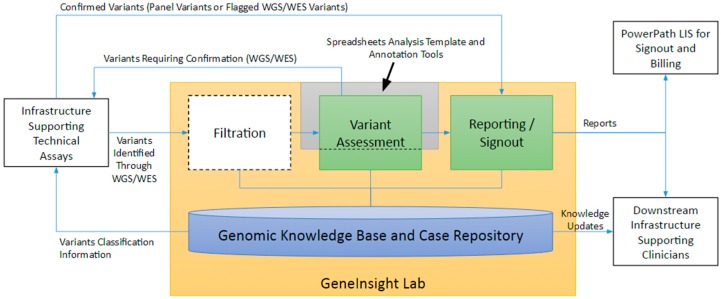
Infrastructure Supporting Interpretation.

The laboratory’s goal in genetic testing is to produce an informative report for the patient’s clinicians that are both clear and concise. To do so, the laboratory must determine which variants warrant inclusion in the report, assess their association with disease or pharmacogenomic effects, and draft a report explaining the potential impact of the variants to the specific patient. The laboratory should then keep the clinicians up to date if new information emerges on a previously reported variant. Establishing and maintaining a knowledge base and case repository infrastructure is fundamental to properly supporting not only these activities but upstream and downstream processes as well. The LMM uses GeneInsight Lab [3] in combination with annotation tools and a spreadsheet-based analysis template to perform these functions (Figure 4). Other systems involved in the interpretation space include Cartagenia (Cartiagenia, Leuven, Belgium) and PierianDx (PierianDx, St. Louis, MO, USA).

### 4.2. Genetic Knowledge Base and Case Repository

The core of our interpretation infrastructure is a knowledge base and case repository that manages information and relationships between tests, genes, variants, diseases and pharmacogenomic effects, and literature references. This infrastructure has been previously described in detail [3], with critical components reiterated here. Case information is validated against and linked to these knowledge base entries. The most important interrelationship is the linking of variants to diseases and pharmacogenomic effects through interpretations that contain classifications of the type and level of certainty of the relationship—for example, Resistant/Responsive, Pathogenic/Benign, or Uncertain Significance. Also critical to associate with each interpretation are the evidence and analysis these assessments are based upon, who approved them, and the reason for any changes.

Another key interrelationship is the management of reference sequence data throughout the system. Transcripts with reference sequence information are associated with each gene. Each test has information on the regions of interest (ROI) it targets. These ROIs are defined relative to the gene’s reference sequence or to a particular genomic location. Variants are validated against the associated reference sequences, providing proper genomic context and nomenclature. Variants associated with cases are also validated to ensure they fall within the test ROI for that patient.

The knowledge base contains workflow and role based security surrounding critical elements. For example, we have configured our instance of GeneInsight Lab to allow multiple roles to propose new interpretations but only geneticists to approve them. The history of variant interpretations and approvals, along with other key data, is tracked and available through the user interface.

GeneInsight Lab allows the knowledge base and case repository to be searched along multiple dimensions. The deep linkage between the case repository and the knowledge base allows the user to search the case repository leveraging information stored in the knowledge base. It also allows drill downs from case repository search results into knowledge base entries. For example, a user can search for cases with variants in a gene of interest and then click on a variant to be brought to its knowledge base entry. From there the user might click on a disease associated with the variant to be brought to its entry. This functionality has proven critical for both routine operations use and translational research studies.

Since the original publication referenced above, numerous features have been added and/or enhanced including support for curation of gene-disease associations, the ability to share variant- and gene-disease associations with other laboratories and the capability to store variant assessment documentation. Support for structural variants has also been expanded.

### 4.3. Filtration

Clinical laboratories running complex molecular testing generate many variants per patient, a number that increases significantly with the advent of exome and genome clinical tests. A strategy to filter out variants that most likely do not impact disease, as well as help prioritize the variants that may be relevant to an indication for testing, needs to be implemented. To achieve this, laboratories must filter these variants in many different ways using different types of data. A few example filters include: loss-of-function variants, frequency in the general population below a certain threshold, or variants not already classified in the knowledge base as Benign or Likely Benign. To enable these tasks, we added a big data technology-based tool to GeneInsight that allows laboratories to flexibly load and store large data sets of variant annotations, separately load and store variants identified in each patient, hybridize the annotations to the patient variants, and then filter variants based on the combined data. Users can create re-usable filter trees that chain together different criteria to identify potentially clinically meaningful variants. These trees can then be stored and repeatedly applied across cases. Patient variants can also be filtered on an ad hoc basis using any combination of annotations that have been previously loaded. The final filtered variant lists are locked and maintained for capturing the exact clinical scenario used in the patient assay.

### 4.4. Variant Assessment

Variants may require a comprehensive assessment as a result of being identified and prioritized through the applied filtration process. All completed variant assessments are stored within the GeneInsight knowledge base. GeneInsight tracks the variant classification relative to disease or pharmacogenomic effect, the evidence behind this classification, and an extensive audit log of the events behind this classification. Reassessment of previously identified variants are only required if there is reason to believe that new potentially valuable information has emerged. The variant assessment process itself is currently spreadsheet based. We maintain a spreadsheet template that organizes the information we consider important for variant assessment to ensure consistency between assessments [10]. The spreadsheet is integrated with GeneInsight and other annotation tools including Alamut (Interactive Biosoftware, Rouen, France) to gather the data needed for interpretation.

We have kept the assessment process spreadsheet based because it enables us to easily modify, evolve and expand the types of data we consider without needing to version the application infrastructure to do so, functionality that was critical as clinical genetics rapidly expanded. The tool was implemented in 2010 prior to clinical NGS and the expansion of resources for interpreting variants. With the recent release of ACMG guidelines [11] or standardizing interpretation of sequence variants and the additional efforts of large-scale consortiums like the ClinGen [12] initiative to develop variant assessment infrastructure, we anticipate the emergence of tools for efficient robust interpretation. We intend to closely monitor these efforts to determine whether we should integrate them into our systems and workflow. These applications may eventually enable us to retire our spreadsheet templates.

### 4.5. Reporting and Sign out

GeneInsight automatically drafts reports for genetic counselor and geneticist review, as previously described in detail [3]. The system drafts these reports based on templates maintained by genetic counselors. Each template has a set of logical conditions associated with it that specify clinical situations where it can be used. Genetic counselors use this feature to create templates that identify and handle different kinds of clinical scenarios. The template with the most specific set of conditions that match the case is chosen by default. Users can then override this selection by choosing a different template. Genetic counselors can also associate boolean conditions with content within a template. In these situations, the content is only displayed if the conditions are met. Genetic counselors can also specify parts of the template that should be populated from the knowledge base or information received from the case.

When GeneInsight drafts a report for a patient, it combines information on the case and within the knowledge base to: (1) select a template, (2) execute the boolean logic within the template to determine which pieces should be retained, and (3) fills in any dynamic sections to produce the complete draft. This draft must then be reviewed and potentially modified by the genetic counselor and ultimately geneticist before it is approved and finalized.

GeneInsight integrates with PowerPath to facilitate report finalization and billing. The GeneInsight Lab infrastructure also communicates with GeneInsight Clinic infrastructure to deliver reports and knowledge updates to clinicians.

## 5. Cross Institutional Sharing

Nearly every element of this process flow either benefits, or in the future could benefit, from cross-institutional data sharing. We currently share variant interpretations both by submitting them to ClinVar [13] and through the GeneInsight VariantWire real-time sharing network. We are currently reviewing potential mechanisms for sharing case level data while protecting patient privacy. Sharing of gene interpretations, which can be facilitated through GeneInsight, and working oligo definitions would also be useful.

## 6. The Future

Going forward, we see a need to further connect clinicians, geneticists, patients, and laboratories with each other, and with the genetic data and other results and phenotypes in the electronic health record and clinical systems. Patient data used in clinical practice is currently very siloed. Geneticists usually do not have access to clinical data that exists in the EHR, radiology, or other clinical systems and are dependent upon limited information filled out in paper requisition forms. Structured, complete data would be extremely useful in assessing the implications of variants, ultimately improving patient care. On the other hand, clinicians often do not have access to the genetic knowledge bases and case histories stored in systems like GeneInsight. This also hampers their ability to deeply assess clinical scenarios as they emerge. There is a need for an application that interfaces with the range of systems that store patient data and organizes these data into unified patient and population views for clinical and laboratory personnel. These views would ideally be disease specific and as comprehensive as possible. Such a system could provide the basis for enhanced clinical decision support infrastructure. It could also facilitate and fundamentally improve laboratory-clinician communication and by doing so improve care for patients.
